# Dual-Level Capacitive Micromachined Uncooled Thermal Detector

**DOI:** 10.3390/s19245434

**Published:** 2019-12-10

**Authors:** Hani H. Tawfik, Karim Allidina, Frederic Nabki, Mourad N. El-Gamal

**Affiliations:** 1 Department of Electrical and Computer Engineering, McGill University, Montreal, QC H3A 0G4, Canada; mourad.el-gamal@mcgill.ca; 2 MEMS Vision Intl. Montreal, QC H1Z 2K4, Canada; karim.allidina@mems-vision.com; 3 École de Technologie Supérieure (ETS), Montreal, QC H3C 1K3, Canada; frederic.nabki@etsmtl.ca

**Keywords:** thermal detectors, MEMS, bimorph, microcantilever, surface micromachined, capacitive sensor

## Abstract

This paper presents a novel dual-level capacitive microcantilever-based thermal detector that is implemented in the commercial surface micromachined PolyMUMPs technology. The proposed design is implemented side-by-side with four different single-level designs to enable a design-to-design performance comparison. The dual-level design exhibits a rate of capacitance change per degree Celsius that is over three times higher than that of the single-level designs and has a base capacitance that is more than twice as large. These improvements are achieved because the dual-level architecture allows a 100% electrode-to-detector area, while single-level designs are shown to suffer from an inherent trade-off between sensitivity and base capacitance. In single-level designs, either the number of the bimorph beams or the capacitance electrode can be increased for a given sensor area. The former is needed for a longer effective length of the bimorph for higher thermomechanical sensitivity (i.e., larger tilting angels per degree Celsius), while the latter is desired to relax the read-out integrated-circuits requirements. This thermomechanical response-to-initial capacitance trade-off is mitigated by the dual-level design, which dedicates one structural layer to serve as the upper electrode of the detector, while the other layer contains as many bimorph beams as desired, independently of the former’s area.

## 1. Introduction

Thermal detectors, also known as bolometers, have a wide range of applications in both the military and civilian sectors. Since their discovery in the 18th century, numerous technologies have been invented for implementing infrared sensors. After World War II, the need for night vision was found to be critical for military purposes, resulting in the development of photon detectors [1]. The emergence of micro-electro-mechanical system (MEMS) technologies in the 80s enabled the potential for microbolometers with reduced cost, without sacrificing performance. Commercial efforts resulted in the implementation of Vanadium Oxide-based microbolometers [2], and pyroelectric arrays [3].

These two technologies still dominate the market of high-performance MEMS infrared (IR) sensors. Although microbolometers have lower cost than photon detectors, they are still considered to be too expensive for medium, low, and ultra-low-end civilian applications such as industrial maintenance, automotive, and portable devices. This limits the expansion into these potentially very large markets [4,5].

In order to reduce the cost of microbolometers, microcantilever thermal detectors were first introduced in [6]. The working principle is based on building a bimorph beam with a thermal expansion coefficient (TEC) mismatch. The rise of the temperature of the beams results in a downwards bending of the structure’s head. This out-of-plane motion can be sensed optically [7,8,9], or electrically through a piezoresistive material [10]. The latter suffers from temperature sensitivity concerns since the piezoresistive effect is inherently temperature dependent [11], while the former requires a bulky optical readout that is not suitable for emerging applications that need to be compact and low-cost [12]. A proposed third readout mechanism was introduced in [13], where the structure’s head movement is measured capacitively. Capacitive MEMS sensors and actuators prove efficient in many sensing applications such as inertial, ultrasonic, and magnetic sensors [14,15,16]. Additionally, capacitive sensors do not exhibit Johnson and 1/*f* noise, which are associated with resistive microbolometers [2].

Microcantilever thermal detectors face many implementation challenges such as susceptibility to shock, vibration, ambient temperature changes, and high residual stresses. Several solutions to overcome these challenges have been reported in [17,18,19]. However, an unaddressed design challenge is the trade-off that arises when expanding the number of adjacent bimorph microcantilevers or beams in a folded structure in order to increase bending displacement and, hence, sensitivity. In this case, the detector head needs to be reduced in size to accommodate the bimorph beams in order to maintain a constant sensor area. This works against the improved sensitivity and reduces the impact of the increased number of folded beams. Here, a novel architecture is proposed to overcome this trade-off by utilizing a two structural layers process to create a dual-level sensor in which the bimorph beams are present above the detector head, and hence are decoupled from its area. This technique allows for maximizing the head area and the bimorph beam area simultaneously.

This paper first reviews the working principle of capacitive micromachined thermal detectors in Section 2.1. Section 2.2 describes the design trade-offs, and the dual-level sensor architecture is introduced in Section 3. Section 3.2 compares the proposed design to four different designs, inspired by the literature, that were implemented alongside the dual-level sensor using the same commercial PolyMUMPs process. Section 3.3 explains how the proposed design also reduces the residual stresses issue typically found in microcantilever thermal detectors. The fabrication process for the devices is described in Section 4, and the characterization setup and measurement results are presented in Section 5.

## 2. Design and Simulations

### 2.1. Working Principle

**Figure 1:**
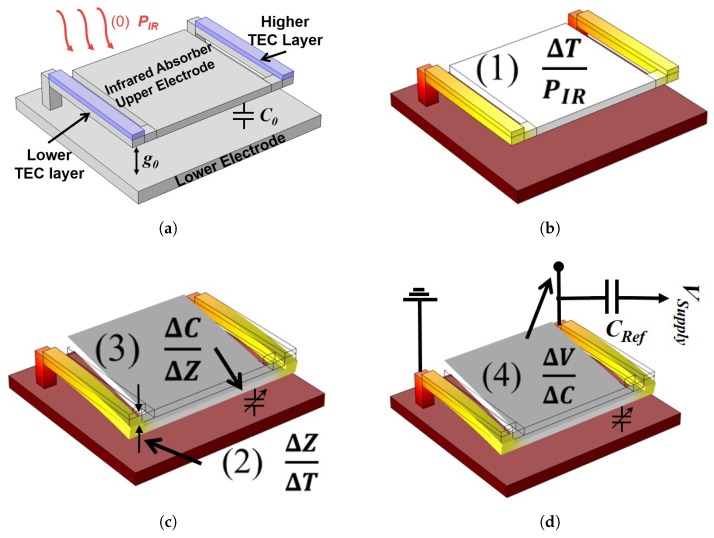
Energy transformations occurring for infrared detection. (**a**) Infrared power ($P_{IR}$) falling on the microcantilever thermal detector. (**b**) The absorbed radiation increases the temperature of the detector by $\frac{\Delta T}{P_{IR}}$. (**c**) The rise of temperature bends the bimorph microcantilever downwards by $\frac{\Delta Z}{\Delta T}$ and consequently changes the detector’s capacitance by $\frac{\Delta C}{\Delta Z}$. (**d**) The change of capacitance results in a change of the output voltage $\frac{\Delta V}{\Delta C}$.

Thermal detectors convert input infrared radiation into an electrical output signal (voltage in this work) as shown in Figure 1. Henceforth, several transformations of energy occur in the process, such that the change in the output voltage of the device is given by:

(1) $$\Delta V=P_{IR}\cdot \frac{\Delta T}{P_{IR}}\cdot \frac{\Delta Z}{\Delta T}\cdot \frac{\Delta C}{\Delta Z}\cdot \frac{\Delta V}{\Delta C},$$

where ΔV is the change of the output voltage, $P_{IR}$ is the infrared power absorbed by the detector’s head, $\frac{\Delta T}{P_{IR}}$ is the change of the detector’s temperature due to the falling infrared, $\frac{\Delta Z}{\Delta T}$ is the deflection of the detector’s upper electrode per degree Celsius, $\frac{\Delta C}{\Delta Z}$ is the change of the detector’s capacitance with respect to the upper-electrode’s deflection, and $\frac{\Delta V}{\Delta C}$ is the change of the output voltage with the change in capacitance. Firstly, a heated source at room temperature transmits electromagnetic radiation in the infrared spectrum that is collected by the thermal detector, as seen in Figure 1a. The collected radiation $P_{IR}$ has a power that is given by [20]:

(2) $$P_{IR}=\eta_{S}t_{L}\left(\frac{A_{D}}{2\pi L^{2}}\right)A_{S}\eta_{D}\sigma_{S-B}\left(T_{S}^{4}-T_{D}^{4}\right),$$

where $t_{L}$ is the transmission of the collecting lens, $A_{D}$ is the detector’s effective area (i.e., the infrared absorbing part which is referred to as the head or upper electrode in this work), $A_{S}$ is the area of the target (source of the IR), *L* is the distance between the target and the detector, $\sigma_{S-B}$ is the Stefan–Boltzmann constant, $T_{S}$ is the target’s temperature, and $T_{D}$ is the detector’s surrounding temperature. $\eta_{S}$ and $\eta_{D}$ are the target’s and detector’s IR absorber emissivities respectively. Due to the direct proportionality that is shown, research efforts were focused on enhancing the emissivity of the used infrared absorbers by using thin metal films [21], quarter wavelength absorbers [22], or gold-black coatings [23].

The IR absorption results in a heating of the structure, as seen in Figure 1b, with a resulting temperature difference that is directly related to the detector’s thermal resistance $R_{thermal}$ such that:

(3) $$\frac{\Delta T}{P_{IR}}=R_{thermal}\approx \frac{1}{G_{conduction}}=\frac{L}{(\lambda_{1}t_{1}+\lambda_{2}t_{2})W},$$

where λ, *t*, and *W* are the thermal conductivity, thickness, and width of the microcantilever, respectively. The subscripts 1 and 2 stand for the higher TEC layer (metal, Gold in this work) and the lower TEC material (typically silicon-based, polysilicon in this work), respectively. This inverse relationship favors the use of more thermally isolated structural layers to increase the thermal isolation of the thermal detector and enhance its overall response. Typically, in deep vacuum conditions, the thermal conductance $G_{conduction}$ of the detector’s beams are the dominant factor. Leakages due to radiation and convection through the air are ignored as they have minimal effects.

Secondly, the rise in temperature results in the bending of the supporting bimorph beams due to the TEC mismatch of the composed layers. This deflection, shown in Figure 1c, can be calculated according to [24]:

(4) $$\frac{\Delta Z}{\Delta T}=2\left(\alpha_{1}-\alpha_{2}\right)\left(\frac{t_{1}+t_{2}}{t_{2}^{2}K}\right)L^{2},$$

where α is the material’s TEC, *L* is the length of the bimorph microcantilever, and *K* is given by:

(5) $$K=4+6n+4n^{2}+\phi n^{3}+\frac{1}{\phi n}$$

where *n* and ϕ are the thickness and Young’s modulus ratio of the bimorph layers, respectively. The higher the TEC mismatch, the greater the deflection will be. A typical choice of materials has been a silicon-based layer such as SiN, SiO${}_{2}$ [25], or SiC [13] due to their low TEC and good mechanical properties, and a metal layer such as Gold (Au) [13] or Aluminum (Al) for their high TEC. Designing the thickness of the layers is not straightforward since it cannot be deduced intuitively from (4) due to its interlacing relationship with the material’s Young’s modulus. However, work in [24] demonstrated that an optimum thickness ratio can be found to maximize the thermal response by calculating the thermal response from (3) with respect to *n*.

**Figure 2:**
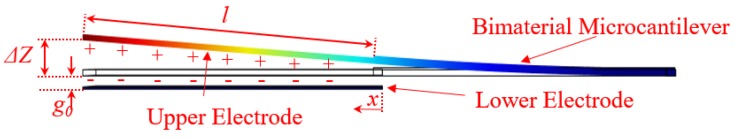
Side-view of a microcantilever thermal detector illustrating the tilting capacitance.

Thirdly, the displacement ΔZ results in a change of the parallel plate capacitor gap between the thermal detector head and the lower electrode lying on the substrate beneath it. The capacitance of a simple parallel plate capacitor can be calculated according to:

(6) $$C=\frac{\epsilon_{0}wl}{g_{0}+\Delta Z},$$

where *C* is the capacitance of the thermal detector, $\epsilon_{0}$ is the permittivity of the vacuum, *w* and *l* are the width and length of the plate, $g_{0}$ is the initial gap distance between the two plates, and ΔZ is the out-of-plane displacement at the tip of the microcantilever (from (4)) due to bending. However, the detector-head (upper electrode) does not displace leveled out-of-plane in a piston fashion; rather, it behaves in a more inclined manner, as seen in Figure 2. Equation (7) considers this inclination by calculating the capacitance on an infinitesimal section of the plate dx then integrating this across the plate length *l* to obtain the total capacitance such that:

(7) $$C=\int_{0}^{l}\frac{\epsilon_{0}wl}{g_{0}+\Delta Z\frac{x}{l}}dx=\frac{\epsilon_{0}wl}{g_{0}}\ln\left(\frac{g_{0}+\Delta Z}{g_{0}}\right).$$

Hence, by differentiating (7) with respect to ΔZ the third term in (1) can be derived as (8):

(8) $$\frac{\Delta C}{\Delta Z}=\frac{\epsilon_{0}wl}{g_{0}}\frac{1}{g_{0}+\Delta Z}.$$

It is worth noting that in this work the high TEC material is placed on top of the bimorph microcantilever, leading the beam to bend downwards, hence, ΔZ has negative values.

Finally, the thermal detector (behaving as a variable capacitor) is connected to a reference capacitor and the fourth term $\frac{\Delta V}{\Delta C}$ in (1) is evaluated depending on the read-out integrated circuit used. In this work, a ZDMI ZSSC3123 capacitance to digital converter (CDC) was used to measure the capacitance (i.e., thermal response) of the fabricated sensors. The CDC output is proportional to the ratio of the measured sensor capacitance with respect to a reference capacitor.

### 2.2. Typical Trade-Off: Sensitivity vs. Area

**Figure 3:**
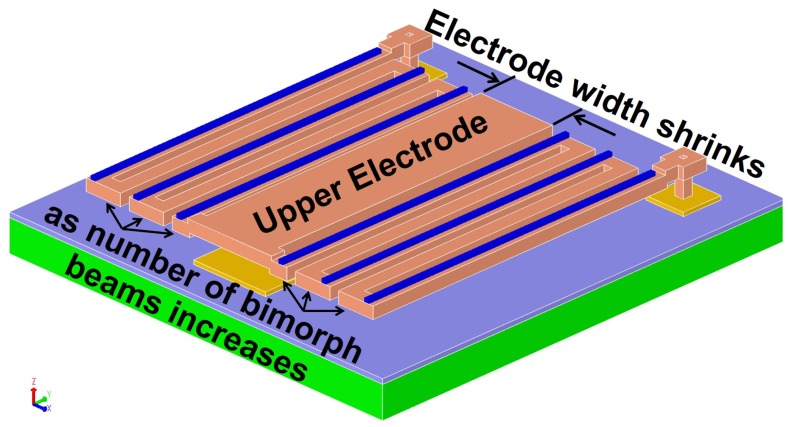
3D model illustrating the trade-off in traditional single-level thermal detectors.

Conventional microcantilever-based thermal detectors reported in the literature are typically single-level, in which the detector’s head (composed of the upper electrode and the IR absorber), and the thermal sensitive bimorph microcantilevers or beams are implemented on the same plane. This creates a trade-off between the number of beams used to increase the sensitivity of the detector and the area of the device. As shown in Figure 3, a longer bimorph beam (larger *L* in (4)) is needed to achieve larger out-of-plane displacement per 1 ${}^{\circ }$C change. One method of increasing the length of the beam while keeping the footprint small is to use multi-fold beams. However, for a fixed footprint area in single-level devices, using a larger number of folded beam sections means the head area of the detector $A_{D}$ must be decreased. This is undesirable, since the initial capacitance value and resolution are directly proportional to $A_{D}$ (from (7) and (8), respectively). Moreover, $A_{D}$ contributes directly to a higher $P_{IR}$ (2). Therefore, these two contradicting design requirements must be traded off with each other.

## 3. Proposed Design and Finite Element Analysis

### 3.1. Dual-Level Architecture

**Figure 4:**
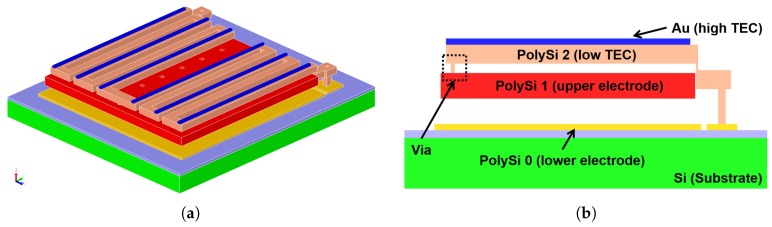
(**a**) 3D view of the proposed dual-level design. (**b**) Cross-section view.

This work attempts to mitigate the previously discussed trade-off by introducing a dual-level thermal detector as depicted in Figure 4a. As shown in the cross-section of the design shown in Figure 4b, the upper level contains as many folded beams as possible (within fabrication process capabilities) to increase the deflection amount with respect to temperature. Below the bimorph beams, the lower level utilizes the entire detector footprint area as the sensor’s upper electrode to maximize the sensor’s capacitance.

The electrical/mechanical connection between both layers is achieved by creating a via hole in the second sacrificial layer of the upper polysilicon (PolySi 2)), allowing it to contact the bottom PolySi 1. This transfers the deflection of the bimorph beams (blue/brown), due to the thermal energy to the upper electrode (red) forming the capacitor. All three PolySi layers (yellow, red, and brown) are highly doped with phosphorous, guaranteeing an electrical path to the upper electrode. The high TEC metal layer was deposited on every beam section to ensure an accumulation of the tilting motion as described in [17].

A focal plane array (FPA) with a high fill-factor is desired to achieve maximum utilization of the inbound infrared power. A method for enhancing the fill-factor of capacitive microcantilever-based thermal detectors was reported in [25], but despite their relative success in achieving better fill-factors than typically reported structures in the literature, the authors’ approach was still limited by the previously explained trade-off, because their design was single-level based.

### 3.2. Reference Designs

From the discussion in Section 2.1, it is clear that many aspects affect the performance of thermal detectors, such as the properties of the materials used and the critical dimensions, many of which depend solely on the fabrication process used. Hence, meaningful comparisons of design-to-design performance are needed in order to characterize the impact of the proposed dual-level structure. Accordingly, in order to evaluate the proposed design, this work also implemented 4 designs inspired by previously reported works in the literature. These were built directly beside the proposed duplex architecture, and therefore used the same materials and critical dimensions. Henceforth, all simulation and measurement results are reported for these 4 designs and the proposed design in order to fairly characterize the performance of the dual-level architecture.

**Figure 5:**
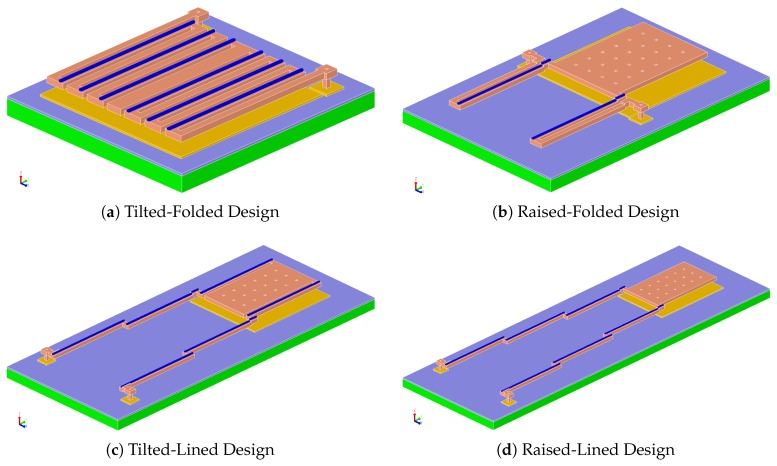
Reference designs implemented for performance comparison.

**Figure 6:**
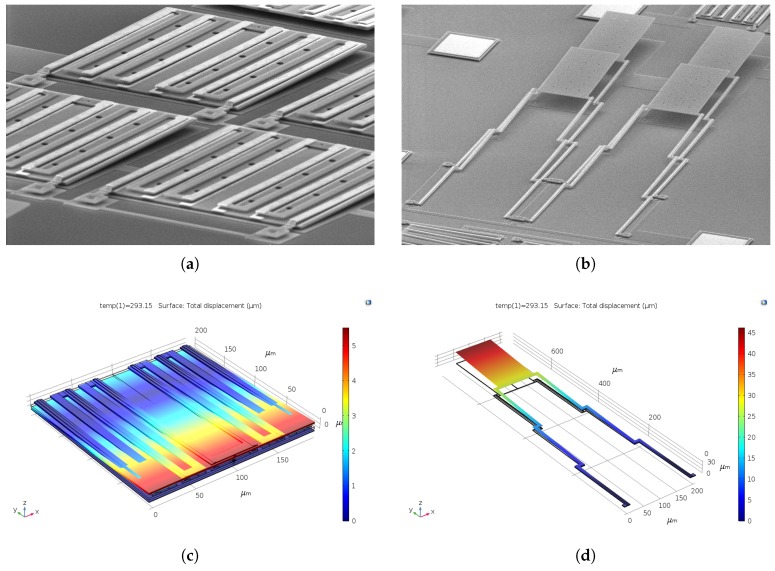
SEM photos of (**a**) a tilted-folded array; (**b**) a raised-lined array. (**c**) FEA showing the intrinsic stresses effect on a tilted-folded design. (**d**) FEA showing the intrinsic stresses effect on a lined–raised design.

The 4 designs chosen can be categorized by two main aspects. The first relates to the way the multi-microcantilever beams are formed. This is either done by folding the beams right next to each other, as in Figure 5a,b, or by *lining* them right after each other, as shown in Figure 5c,d. Arrays for the tilted-folded designs have adjacent sensors placed *next to* each other, as depicted in Figure 6a, while for the rest different pixels are tiled *inside* each-other (e.g., see Figure 6b), allowing the use of longer microcantilevers with a stair-like structure. It is worth noting that tilted-folded and raised-folded designs require the use of at least double the number of beams when compared to the tilted-lined and raised-lined designs for approximately the same deflection rate per degree Celsius. This is due to the requirement of placing a unimaterial beam within every two bimorph beams to ensure an addition of the tilting angles [17]. Therefore, the tilted-folded and raised folded designs suffer from a further reduction of the pixel-head area $A_{D}$.

The second categorization aspect emerges from the fashion in which the detector head is displaced with temperature. Either the head *tilts* around its center when the beam bends, as in the designs shown in Figure 5a,c, or the entire head is *raised* above its nominal level, as is the case in the designs shown in Figure 5b,d.

A finite element analysis (FEA) was performed on the 5 sensors using the COMSOL Multiphysics simulation suite. As shown in (1), 5 different aspects need to be taken into account for a full analysis. This requires multiphysics simulations in which the thermo-electro-mechanical physics are accounted for simultaneously. Due to the heavy computation requirements of such simulations, only a thermo-mechanical simulation was performed. Table 1 summarizes the critical dimensions of the 5 designs and the simulation results of the deflection rate and stress-induced tilt deflection.

### 3.3. Residual Stress Considerations

The unmatched residual stresses of Au and PolySi 2 in the bimorph beams lead to a significant initial curvature upwards that reduces the sensor’s capacitance. A further study on the effective height of bimorph microcantielvers implemented in PolyMUMPs is reported in [26]. It is even possible for this capacitive reduction to reach levels that necessitate the use of thermoelectro (TE) coolers for compensation [27]. A TE cooler in a sensor package adds cost, size, and power consumption, so eliminating the need for one is desired. Hence, it is of paramount importance to fabricate a structure with reduced stress.

The tip deflection due to residual stresses was simulated using FEA with COMSOL Multiphysics and the results are included in Table 1. Only the solid-mechanics physics are considered. Average values of the initial PolySi 2 and metal stresses and thermal expansion coefficients were obtained from the PolyMUMPs design rules [28]. It can be noticed from the simulation results that designs with *folded* beams suffer less from residual stress than those incorporating lined beams. This can be explained by examining (9) [26], which gives the amount of vertical tip deflection *h* (for a single bimorph beam) due to residual stress, causing an angle θ=L/ρ of the arc spanned by the microcantilever as

(9) $$h=\rho \left[1-cos\theta \right]=\rho \left[1-cos\left(\frac{L}{\rho }\right)\right]\approx \frac{L^{2}}{\rho }.$$

If *i* instances of *lined* bimorph beams are used, they will approximately act as one beam of length iL, leading to a total deflection approximately proportional to $i^{2}h$. Hence, extra measures are needed to mitigate residual stresses. Zhao et al. in [7] reported an initial bending of 15 μm for 200 μm long SiN-Au, *lined*, bimorphs. To reduce the bimorphs’ initial bending, two layers of SiN were deposited on top of each other with different thickness and stoichiometry which reduced the initial bending down to 4–5 μm. Furthermore, the authors found it necessary to heat the sensors above room temperature up to 85 ${}^{\circ }$C, to further reduce the initial upward bending for better performance.

In comparison, the curvature angle θ=L/ρ for the *folded* case is added for every fold [8,29]. Hence, a total curvature angle of iθ leads to a deflection that is directly proportional to ih. The FEA results in Table 1 support this hypothesis. Hence, a folded beams based device was chosen in the proposed dual-level design. Inspection of the SEM images of fabricated devices shown in Figure 6a,b and comparing them to their corresponding FEA results shown in Figure 6c,d, respectively, validates the conclusion proposed earlier here.

## 4. Fabrication

**Figure 7:**
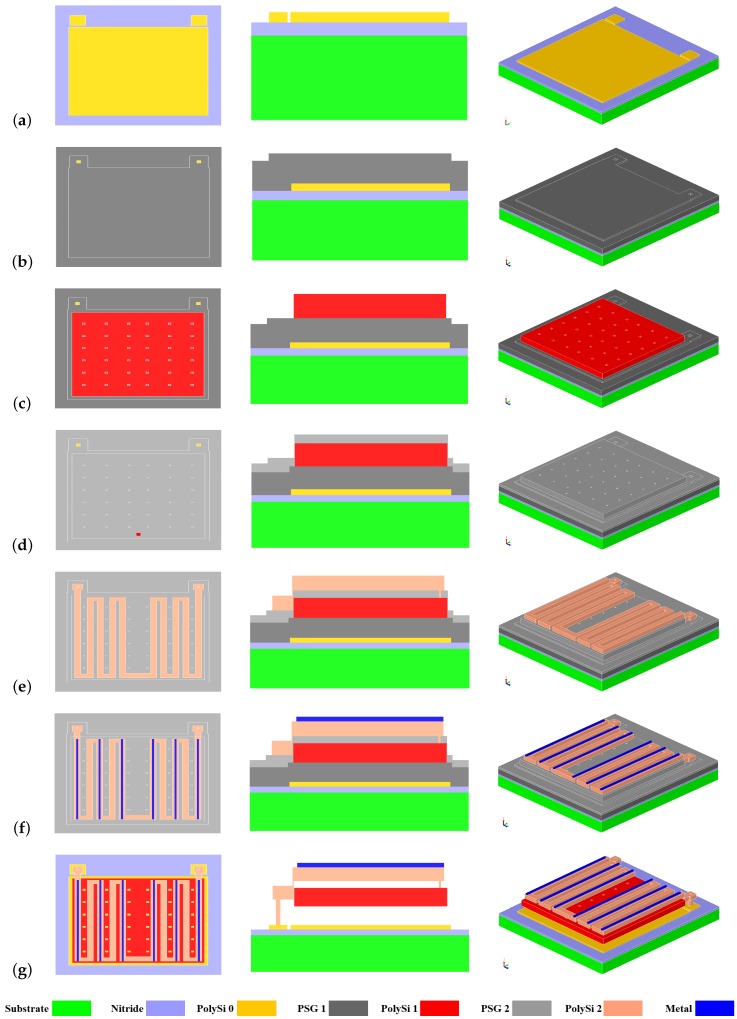
Fabrication process flow of the proposed dual-level design. (**left**) Top-view. (**middle**) Cross- section. (**right**) 3D isometric view.

All structures were fabricated using the PolyMUMPs platform provided by MEMSCAP. The surface micromachined process consists of three phosphorus doped PolySi layers. Figure 7 depicts the utilization of this platform for implementing the proposed dual-level design. The illustration consists of three views (top, cross-section, and isometric) for the major steps in the process. The process is built on a low-resistance silicon (Si) substrate (1–2 Ω·cm resistivity) with an insulation layer of 600 nm silicon nitride (SiN) deposited using a low-pressure chemical vapor deposition (LPCVD) process. Then, the first LPCVD PolySi 0 layer of 0.5 μm thickness was deposited and patterned to function as the lower electrodes and used for electric signal routing (Figure 7a). After that, the anchors of the dual-level sensor were defined in a sacrificial layer of 2 μm LPCVD phosphosilicate glass (PSG) (Figure 7b). Next, the second LPCVD PolySi 1 layer of 2 μm thickness was deposited to form the first level of the dual-level sensor (Figure 7c). This layer typically would have many purposes in traditional designs, while in the proposed design it serves only as an upper electrode for the capacitive sensor. Structural layers are typically anchored to the substrate, however, in the proposed approach, they are anchored by the bimorph beams, which are deposited afterward, as explained in Section 3.1. Hence, while depositing the 2nd sacrificial PSG layer a hole is patterned to form the via (Figure 7d). This via hole is to be filled during the third (and last) PolySi 2 layer deposition which is patterned into the multi-folded beams to implement the second level of the dual-level design (Figure 7e).

It is worth noting that the spacing between the beams is not uniform. This is because the lower level requires a set of holes to help release the structural layer later from the underlying sacrificial oxide layer. Therefore, it is paramount to ensure that these holes are not sealed by the second level. Accordingly, the spacing between the beams is made wider where a release hole is needed. Otherwise, the spacing is set to a smaller value in order to accommodate more beams. Lastly, a 0.5 μm layer of Au is deposited on top of the PolySi 2 beams to form the thermally sensitive bimorph (Figure 7f).

**Figure 8:**
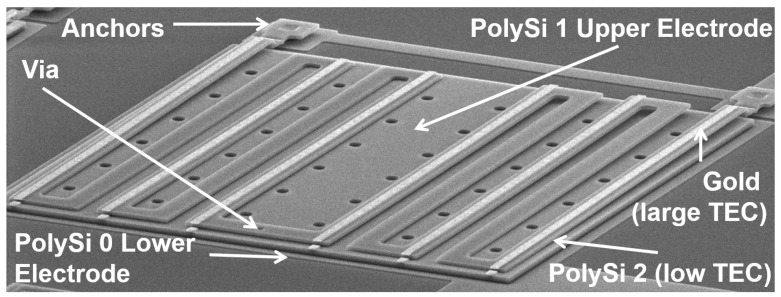
SEM photo of the fabricated dual-level thermal detector.

It can be seen that Au is not fully covering the PolySi 2 beams. That is to abide by the design rules of the process that require a minimum overlap between the PolySi 2 and Au layers. This fact will limit the performance of the sensor, since a width-matched bimorph is more responsive to heat. Finally, the PSG sacrificial layers are etched away in hydrofluoric acid to release the structure (Figure 7g). Figure 8 shows a SEM image after fabrication and highlighting the underlying gap, top folded beam layer, and anchor.

## 5. Characterization and Discussion

**Figure 9:**
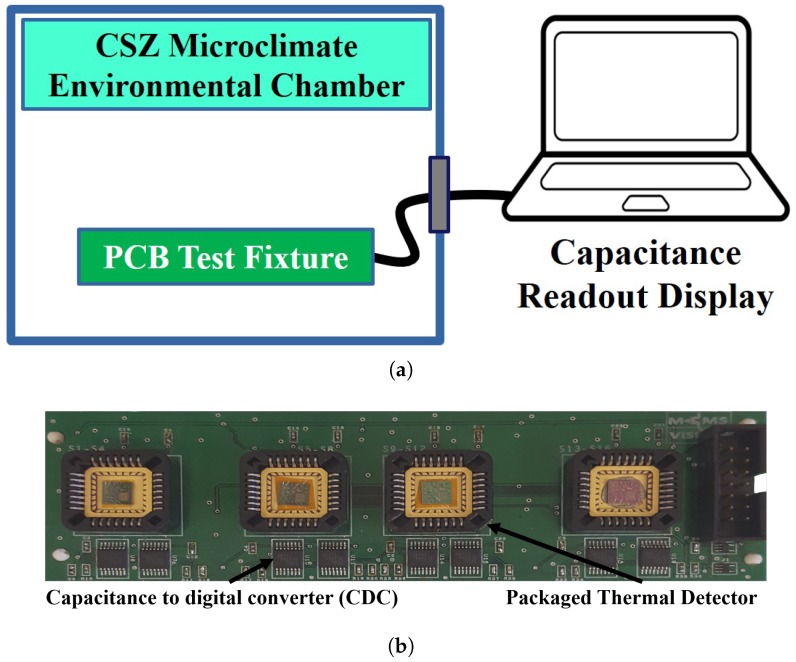
(**a**) Test setup illustration, and (**b**) photograph of the printed circuit board test fixture used for testing.

**Figure 10:**
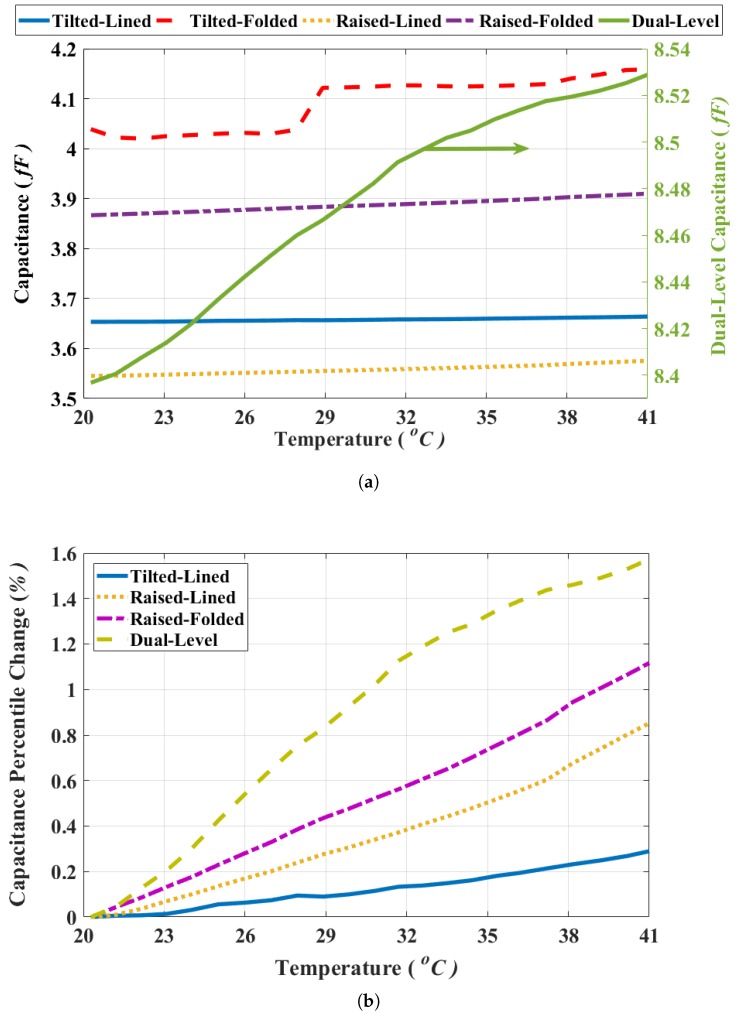
(**a**) Thermal detectors’ capacitance vs. temperature, and (**b**) percentile change of detectors’ capacitances vs. temperature.

Each thermal detector design was wire-bonded using a 28-pin leadless chip carrier (LCC) ceramic package that was mounted on a printed circuit board (PCB) test fixture, as shown in Figure 9. Four identical sensors of each of the 5 designs were measured, and each was connected to the CDC readout integrated circuit. All designs used the same area for the entire detector to provide a representative comparison. The test fixture was then placed inside a CSZ microclimate environmental chamber to measure the change in the capacitance of each sensor with respect to temperature. Importantly, the test setup, as depicted in Figure 9, allows simultaneous measurements of all the devices under the same conditions to achieve an accurate comparison. The humidity inside the chamber was fixed at 30% RH, while the temperature was varied over the range of 20 ${}^{\circ }$C to 40 ${}^{\circ }$C in steps of 1 ${}^{\circ }$C. A custom software program continually collected the temperature of each device and the capacitance readings of the sensors. Figure 10a shows the capacitance measurements of the 5 designs, and Table 2 summarizes their performances. The capacitance change per ${}^{\circ }$C of the dual-level design is three times larger than the next best performing design. Figure 10b plots the percentile change of the different designs’ capacitance vs. temperature, also showing the superiority of the dual-level design. 

The tilted-folded design was not included in the latter plot due to a behavioral malfunction that can clearly be seen from its response in Figure 10a. This strange response is probably due to the large effect of its fringing capacitance that would be comparable to its small parallel-plate one. Despite its better response to bimorph residual stresses, the tilted-folded design was not used with a capacitive readout before in the literature given its unreliable behavior. However, the dual-level approach allowed for a reliable capacitive readout thanks to the addition of the large parallel plate electrode on the bottom level. Moreover, it exhibits a better response to residual stress for an overall higher performance. Table 2 summarizes the figures of merit used for comparing the performances of the different designs, including: (a) the fill-factor, which is the percentage of the detector’s electrode with respect to its total area; (b) the initial capacitance of the different designs at room temperature; (c) the rate of change of the capacitance per degree Celsius; and (d) the temperature coefficient of capacitance (TCC). It is worth noting that the relatively high thermal conductivity of PolySi in comparison to typical alternatives from the literature such as SiN, SiO${}_{2}$, and SiC is responsible for the lower response for all 5 designs compared to the results from the literature. The use of PolySi was necessary on the PolyMUMPs process. However, the proposed dual-level design would benefit equally from the use of an alternate material in a custom process. Since all devices suffered from the same PolySi-related issue, the results presented here highlight the potential of realizing high-end microbolometers if the proposed structure was implemented in a more suitable fabrication process.

**Figure 11:**
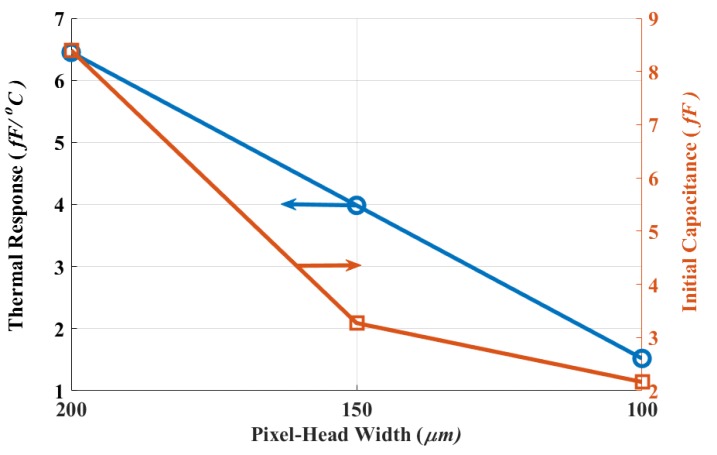
Dual-level design performance for various footprints.

Scaling down the thermal detector footprint area was also characterized by measurements. Figure 11 plots the thermal response and initial capacitance of different detector areas. The inversely proportional relationship to the area for both the thermal response and initial capacitance was expected from (4) and (7) respectively. The proposed design achieves a 1.5 fF/${}^{\circ }$C capacitance change when sized to be only 100 × 100 μm${}^{2}$, and a 6.5 fF/${}^{\circ }$C for a 200 × 200 μm${}^{2}$ design. This shows the potential of the proposed design to minimize area and still perform similarly to the other designs.

## 6. Conclusions

A novel dual-level microcantilever thermal detector design was introduced to improve the trade-off between the thermomechanical response and the initial capacitance. This design was compared to other architectures from the literature by implementing four other typical designs in the commercial surface micromachining PolyMUMPs technology by MEMSCAP, and the proposed design was found to provide superior performance.

The capacitive response with respect to temperature was measured for all five designs and the proposed design exhibited a three-fold improvement in capacitance change per ${}^{\circ }$C. In addition, measurements of the sensitivity vs. the detector area for the proposed design were performed and the results showed that a $100\;\times 100\;\mu^{2}$m thermal detector has a capacitive response of 1.5fF/${}^{\circ }$C. This demonstrates the potential of this architecture to maximize the thermal response of microcantilever thermal sensors for a given area. The superior performance demonstrated here could be further improved by using a structural material with more compatible thermal properties such as SiN.

## Figures and Tables

**Table 1:** Comparison table between the proposed design and several designs inspired from the literature.

	Tilted-Lined	Tilted-Folded	Raised-Lined	Raised-Folded	Dual-Level (Proposed)
**Head Dimensions (μm${}^{2}$)**	200×110	200×20	200×110	200×140	200×200
**Microcantilever Dimensions (μm${}^{2}$)**	200×9	200×9	200×9	200×9	200×9
**Deflection Rate (nm/${}^{\circ }$C)**	339	117	339	39	119
**Tip Deflection Due to Stress (μm)**	27.9	9.7	27.9	3.2	9.8

**Table 2:** Measurement Results Comparison.

	Tilted-Lined	Tilted-Folded	Raised-Lined	Raised-Folded	Dual-Level
**Fill-Factor ${}^{+}$ (%)**	55	10	55	70	100
**Initial Capacitance (pF)**	3.65	4.04	3.55	3.87	8.34
**Rate (fF/${}^{\circ }$C)**	0.5	*	1.4	2.1	6.5
**TCC (ppm)**	135	*	403	535	768

${}^{+}$ Anchors areas are not included. * The capacitance change rate and TCC values were not included due to a behavioral malfunctioning.
